# The Preparation and Dust Suppression Performance Evaluation of Iron Ore Tailing-Based Cementitious Composites

**DOI:** 10.3390/molecules29092056

**Published:** 2024-04-29

**Authors:** Miaomiao Nie, Shefeng Li, Xuli Li, Shenxu Bao, Pei Chen, Yong Zhang, Siyu Ding, Jiale Li

**Affiliations:** 1 School of Chemical and Environmental Engineering, Wuhan Polytechnic University, Wuhan 430023, China; 20230311015@whpu.edu.cn (M.N.); lixuli@whpu.edu.cn (X.L.); chenpei@whpu.edu.cn (P.C.); yongzhang@whpu.edu.cn (Y.Z.); siyu_ding@whpu.edu.cn (S.D.); 20210312004@whpu.edu.cn (J.L.); 2 Hubei Engineering Research Center for Soil and Groundwater Pollution Control, Wuhan 430070, China; 3 Pilot Base of Ecological Environmental Chemicals and Low-Carbon Technology Transformation, Wuhan 430023, China; 4 School of Resources and Environmental Engineering, Wuhan University of Technology, Wuhan 430070, China; sxbao@whut.edu.cn

**Keywords:** iron ore tailings, cementitious material, mechanical properties, wind erosion

## Abstract

In order to comprehensively utilize iron ore tailings (IOTs), the possibility of using IOTs as raw materials for the preparation of cementitious composites (IOTCCs) was investigated, and IOTCC was further applied to mine interface pollution control. The mechanical properties, hydration products, wind erosion resistance, and freeze–thaw (F–T) cycle resistance of IOTCCs were evaluated rigorously. The activity index of iron tailings increased from 42% to 78% after grinding for 20 s. The IOTCC was prepared by blending 86% IOT, 10% ground granulated blast-furnace slag (GGBS), and 4% cement clinker. Meanwhile, the hydration products mainly comprised ettringite, calcium hydroxide, and C-S-H gel, and they were characterized via XRD, IR, and SEM. It was observed that ettringite and C-S-H gel were principally responsible for the strength development of IOTCC mortars with an increase in curing time. The results show that the kaolinite of the tailings was decomposed largely after mechanical activation, which promoted the cementitious property of IOT.

## 1. Introduction

In recent years, mining and smelting processes have resulted in substantial solid waste accumulation. Iron ore tailings (IOTs)—as an inevitable industrial byproduct—have a substantial (and increasing) annual output globally [1]. As per a report, Malaysia produces approximately 625,000 tons of IOT every year. China has the largest tailing stock amount (over 60 billion tons), accounting for more than 40.9% in 2023 [2]. The large stockpiling of IOTs has triggered profound impacts on the environment, safety, and economic fields [3]. On the one hand, IOTs are generally small in particle size and lack organic matter fixation. Harmful components such as heavy metal ions and residual agents are easily released and migrated by mixing rainwater runoff and dust, thereby destroying the local ecological environment [4,5,6]. On the other hand, in order to cope with an increase in tailing stock, the height of a tailings dam also increases; this has gradually become a major potential threat to mine safety production. In addition, the stockpiling of IOTs has resulted in substantial costs for mining enterprises, and these costs are related to land acquisition, construction, and the maintenance of tailings reservoirs. It is reported that the design investment of tailings reservoirs can account for 20–30% of the entire project’s investment [7,8]. However, with the introduction of policies, such as the establishment of a long-term mechanism for the prevention and control of tailings pollution published by the Ministry of Ecology and Environment, the construction cost of tailings ponds continues to increase.

Mechanical activation, chemical activation, and thermal activation are commonly used to improve the activity of cement materials [9,10]. Mechanical grinding was used to improve the reactivity of mining wastes. It could induce changes in the physical and chemical properties of particles, such as particle size, crystal structures, and surface properties [11]. Distortion and dislocation occur during the grain grinding process, which forms a number of activation centers with a decrease in the activation energy of reactions [12]. Marlo et al. [13] studied the influence of mechanical treatment and magnetic separation on the performance of IOTs as supplementary cementitious materials. Even studies using high-crystallinity materials show that IOTs can be used as a supplementary cementitious material. Zhang et al. [14] studied the mechanochemical activation of iron ore tailing-based ternary cementitious materials and concluded that the crystallinity and elemental surface binding energy of an IOT decreased under the action of mechanical activation. The introduction of chemical activators further reduced the crystallinity of an IOT and the surface binding energy of the three elements. In terms of chemical activation, the cementitious properties of the IOT were stimulated through a few reagents. Alkali solutions, including Ca(OH)_2_, KOH, NaOH, and Na_2_SiO_3_, are the most common activators in use. In this manner, the treatment that turns raw aluminosilicate materials into cementitious materials is referred to as alkali activation. Under alkalis, Si-O and Al-O bonds with a high degree of polymerization in the IOT are broken down to form unsaturated bonds, and this accelerates the formation of hydration products [15,16,17]. Thermal activation is a heat treatment process that converts IOTs into pozzolan. The properties of pozzolan are formed via amorphous silica and alumina after calcination. The pozzolanic properties of IOTs are closely related to the activation temperature [18]. Yi et al. [19] confirmed that the lattice distortion was increased in quartz, and the activity of IOT was improved when the activation temperature reached 900 °C. Currently, global research on IOTs mainly focuses on the recovery of valuable elements, gob backfill, soil amendment, the preparation of new building materials, ceramics, glass–ceramics, etc., and they have been considered by many scholars as a secondary resource [20]. The major chemical components of IOTs are silicon dioxide, aluminum oxide, calcium oxide, magnesium oxide, and ferric oxide, which are similar to the chemical components of cement. Therefore, recycling IOTs for the preparation of cementitious materials is an environmentally friendly and economically feasible method [21]. For example, Karthikeyan B et al. [22] used dumped IOTs as a partial replacement in different proportions when waste marble powder is used as a filler material, replacing a smaller constant portion of cement, and the results were compared with traditional concrete. The optimum mix was identified as a combination of 30% IOTs and hybrid fibers. Similarly, Chen Z et al. [23] investigated the application of IOTs instead of natural sand in concrete, and the results indicated that IOTs could significantly improve the performance of concrete. A Saedi et al. [24] studied cementitious materials by replacing cement with 20% lead–zinc tailings while increasing the curing time of samples from 28 to 90 days; the compressive strength increased from 16.9 to 19.8 MPa. There are also some problems in the application of iron tailings with respect to the preparation of cementitious materials. Firstly, IOTs are usually used as a supplementary additive to add to cement materials while their contents generally do not surpass 50%. Secondly, construction bricks prepared using iron tailings have high transportation costs and poor economic benefits. Therefore, developing a new technology to realize the reduction and in situ utilization of iron tailings is urgently needed. The economic efficiency of MT-based geopolymer production depends on the number of costs incurred by the consumers of mine waste for their transportation. In this regard, the territorial proximity of the mining enterprise relative to the planned consumers of MTs and the areas of consumption of finished geopolymer products is of significant importance. With this in mind, this experiment attempts to apply IOT-based cementitious composites (IOTCCs) to dust control in an open-pit mine area. In this paper, the possibility of preparing new cementitious materials using IOTs and their application to mine interface pollution control were studied. The effect of mechanical activation on the activity of iron tailings was studied, and the activated iron tailings powder was used to prepare cementitious materials under the action of small amounts of excipients and an alkali activator. We then sought to apply this process to prevent contact surface contamination in open-pit mining to achieve the goals of iron scrap reduction, resource utilization, and on-site utilization.

## 2. Materials and Methods

### 2.1. Materials

IOTs, ground granulated blast furnace slag (GGBS), and Portland cement were used for mortar production. Na_2_SiO_3_ and NaOH were used as chemical activators to activate IOTs. NaOH (AR, ≥98%) and Na_2_SiO_3_ (AR, ≥98%) were used to promote the hydration process in this study. The IOTs used in this study were obtained from Hubei Daye China. After sieving from a standard square hole screen of 1 mm to eliminate agglomerates and drying at 60 °C in an air-drying oven to remove water, raw IOTs were ground in a planetary mill for 20, 40, 60, 80, 120, 180, 240, 360, and 420 s to obtain different powder samples. Granulated blast-furnace slag was supplied by the Tangshan steel-refining plant, with 0.080 mm sieve residue at 5.0% and a Blaine’s specific surface area of 425 m^2^/kg. Portland cement with a compressive strength of ≥42.5 MPa was cured for 28 d, conforming to the Chinese national standard GB 175-2007 [25].

**Figure 1:**
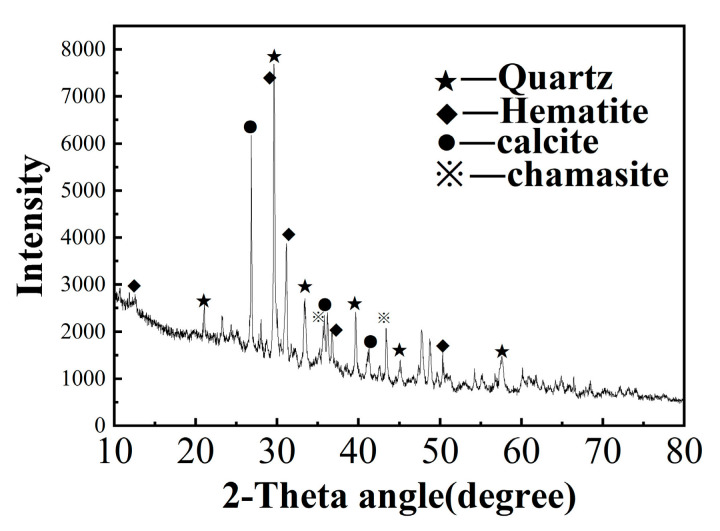
XRD pattern of IOTs.

The raw materials’ chemical compositions are shown in Table 1. The IOTs mainly comprised Fe_2_O_3_ (19.510%), Al_2_O_3_ (7.201), and SiO_2_ (26.113%). The main chemical components of GGBS are as follows: CaO (31.559%), SiO_2_ (27.070%), Al_2_O_3_ (19.958%), and MgO (14.055%). As shown in Figure 1, the main mineral phases of the IOTs comprised quartz, amphibole (including grunerite, kozulite, and cummingtonite), stilpnomelane, and muscovite.

### 2.2. Mix Proportions and Sample Preparation

The IOTs were combined with GGBS and Portland cement via mechanochemical coupling to prepare concrete–mineral admixtures. Mortars were prepared according to the Specification for Mix Proportion Design of Ordinary Concrete (Chinese national standard, JGJ55-2011 [26]). The mix proportion’s design is shown in Table 2. The ratio of water to the binder in all mixtures was kept at 0.45. All samples were cast into cubic moles with dimensions of 40 × 40 × 160 mm^3^ and moved into a curing chamber where the relative humidity and temperature were ≥95% and 25 ± 1 °C, respectively. After 24 h, all specimens were demolded and subject to water submersion for 28 days. Six samples from each group were tested for compressive strength according to GB/T17671-1999 [27]: Method of Testing Cement—Determination of Strength. The mix ratios of the orthogonal experimental design and different water–binder ratios are provided in Table 2 and Table 3.

### 2.3. Test Methods

The particle size distribution was determined via laser diffraction using Master Sizer 2000—Malvern Instruments. The specific surface area and porosity of the material were determined via the gas sorption test, using Quanta Chrome Instruments Nova 1200e (Anton Paar QuantaTec Inc., Boynton Beach, FL, USA). The Braunauer–Emmet–Teller (BET) method was adopted for specific surface tests.

X-ray diffraction (XRD) analysis was performed using Japanese Rigaku Smart Lab SE. The IOT powders under different mechanochemical activations were tested via XRD, which used Cu targets. The X-ray source was operated with the following parameters: voltage: 40 kV; current: 135 mA; range: 10–80° 2θ; step size: 0.017° 2θ; and scanning speed: 2° per min.

The strength index method is used to evaluate volcanic ash. For the common method of assessing the activity of the material, the specific process refers to the standard GB/T 12957-2005 [28]. The calculation of strength index method is shown in Equation (1).

(1) $$K=\frac{R1}{R2}\times 100\%$$

In the formula, K is the compressive strength ratio, that is, the strength activity index; R1 is the 28 d compressive strength of the sample after adding a certain amount of volcanic ash, MPa; and R2 is the 28 d compressive strength of a blank sample of cement, MPa. According to GB/T 12957-2005, the content of pozzolanic material in the experimental samples is 30%.

Normal consistency was used for water requirements, and the setting-time tests of Portland cement and the developed blended types of cement were conducted using a Vicat probe and Vicat needle apparatus. Stability was determined using the Le Chatelier soundness method. The determination of water requirements for standard consistency was necessary for the further application of this material. According to GB/T1346-2001 [27], the standard water consumption consistency of iron tailing-based cementitious materials was determined, including the standard consistency water consumption, initial setting times, and final setting times.

Scanning electron microscopy (SEM) was performed on 3-day and 28-day paste samples. To determine the elemental compositions of the hydration products, roughly 20 points on each paste sample were selected for EDS analysis.

Wind erosion tests were carried out using a wind tunnel. The wind tunnel was fabricated using 2 mm galvanized iron sheets with dimensions of 0.3 × 0.3 × 2.25 m^3^, and it has four windows made of plexiglass. The wind speed in the tunnel reached 23 m/s. These windows were designed for the installation of anemometers and samples, assisting the observation of the wind erosion process. The wind speed was 23 m/s during the wind erosion resistance test. The measurement duration with respect to wind erosion resistance was 5 min.

In three Petri dishes, an equal amount of naturally dried tailings was added, and this was sprayed with test-developed dust suppressant solution A, commercially available dust suppressant B (plant gum with surfactant), and commercially available dust suppressant C (high polymer prepared from plant extracts). The mixture was completely condensed into the wind tunnel, the wind speed was adjusted to 23 m/s, and the blowing time was set to 5 min. The rate of the wind erosion resistance of this dust suppression material was measured. The wind erosion rate is calculated as follows:

(2) $$\upsilon =\frac{G2-G1}{G2}\times 100\%$$

where υ is the wind erosion rate, *G*1 is the mass of the initial tailing soil layer, and *G*2 is the mass of the tailing soil layer after the blowing test. L is the dust suppression rate.

The soil layer after spraying dust suppressant and curing was examined in order to test whether the material can withstand changes in cold and high temperatures relative to different seasons. The dust suppressant was placed in the test mold, and then it was kept at −20 °C and 80 °C for 24 h to form a freeze–thaw cycle—for a total of three cycles—finally, it was removed at room temperature to determine its strength.

## 3. Results and Discussion

### 3.1. Mechanical Activation of Iron Ore Tailings

**Figure 2:**
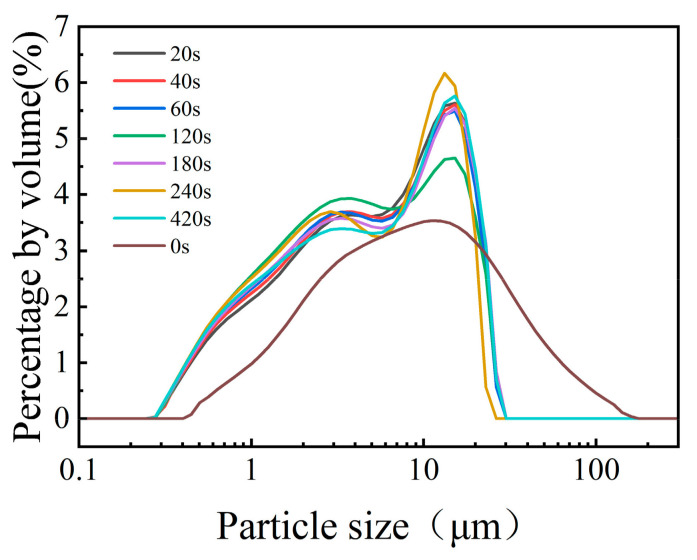
Particle size distribution for different mill times of raw sand materials.

In order to study the effects of mechanical activation on the particle size, specific surface area, and relative crystallinity of IOTs, the mechanical grinding time was set from 20 to 420 s. The cumulative particle diameters (D_90_) and the median particle diameters (D_50_) of iron tailing powder after different grinding times are shown in Table 4. The particle size distribution curves of raw IOTs and the mechanically activated IOTs after grinding for different time periods are shown in Figure 2. The results indicated that the particle size distributions of the mechanically activated IOTs were typically bimodal after milling, with two distinct particle size ranges of 0.5~7.0 μm and 10~25 μm. The D_50_ values of the IOT powders were 5.143 μm and 4.346 μm; the D_90_ values of the IOT powers after grinding for 20 s and 180 s were 16.586 μm and 14.667 μm, respectively. It was reported that the activity could be greatly improved when the particle size of the tailings was less than 20 μm [28,29]. When grinding for 20 s, the iron tailing powder used in this experiment was reduced from 38.547 to 16.588, a decrease of 59.96%. Subsequently, reactivity index analyses were performed to verify this conclusion. Therefore, the vibration grinding time of 20 s was selected.

**Figure 3:**
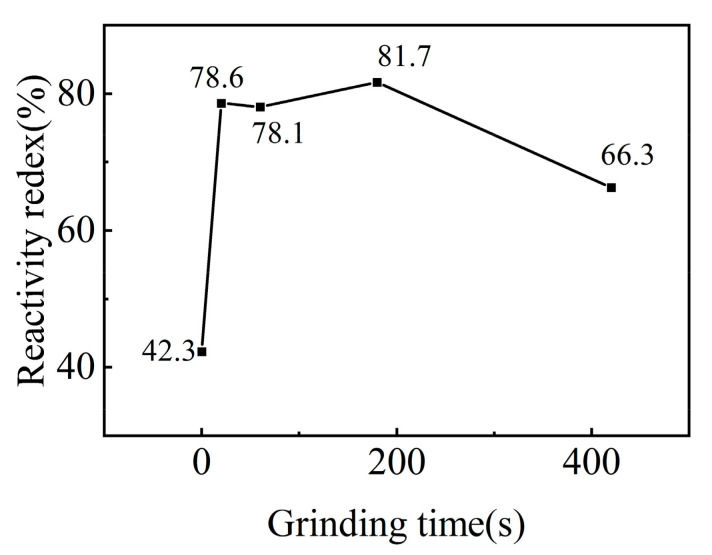
Reactivity index with respect to vibration grinding times.

The potential cementitious activity of iron tailings was activated via mechanical grinding and vibrations. The mechanically activated iron tailings induced a physical filling effect, and they participated in hydration reactions within the cement system. In order to explore the relationship between the reaction index of iron tailings and the grinding time, we further analyzed the reaction activity index according to the national standard GB/T12957-2005. As shown in Figure 3, when the vibration milling time was 20 s, the reactivity index of the iron tailing powder was greatly improved from 42.3% to 78.6%. With a continuous increase in vibration milling time to 420 s, the activity index increased a little. This showed that the iron tailing powder used in this experiment exhibited good grindability and could be broken to a smaller size in a short period of time. Simultaneously, mechanical vibration grinding introduced some mechanical energy to the raw material and increased its reactivity.

**Figure 4:**
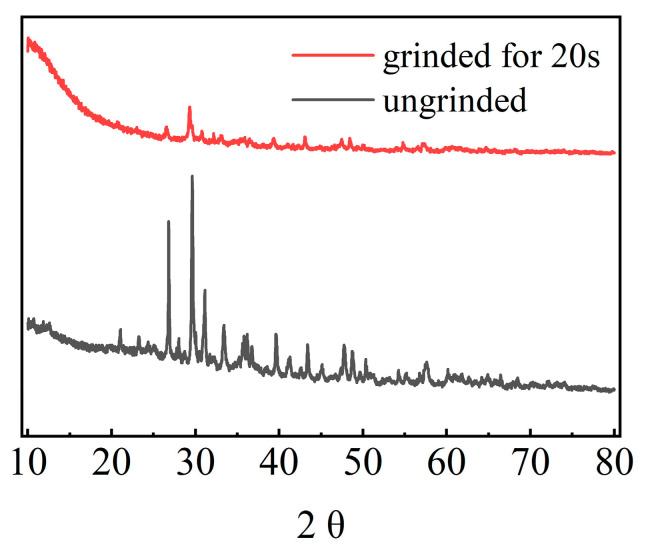
XRD analysis of 20 s of vibrational milling and unground iron tailings.

The XRD patterns of the raw IOT and mechanically activated IOT powders ground at different times are presented in Figure 4. Semi-quantitative estimates of the degree of amorphization of the IOTs were carried out using relative intensities in this study [30,31]. Because grinding energy was applied to the powder, the diffraction peak intensity of IOT powders was decreased. This indicated that increased mechanical grinding reduced the crystallinity of the powder. From the above analysis, all changes revealed that the crystalline structure of the IOTs was gradually changed to an amorphous structure via mechanical grinding.

### 3.2. Mechanical and Physical Properties of IOT-Based Cementitious Composites

**Figure 5:**
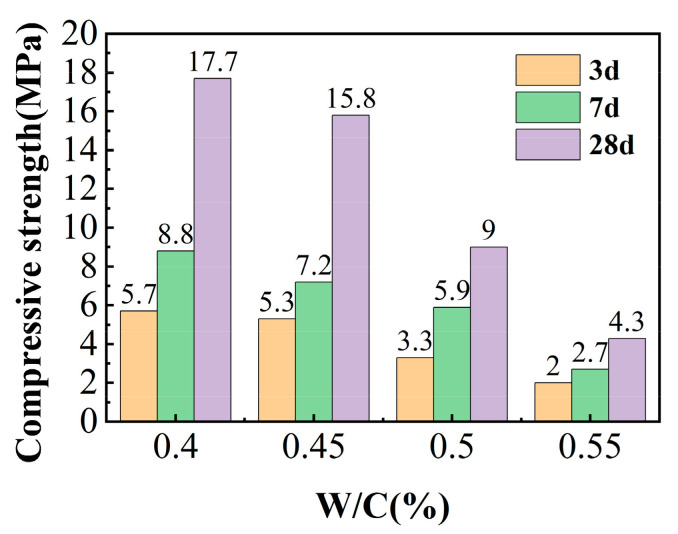
Influence of the water–cement ratio on the compressive strength of test blocks.

The influence of different water–cement ratios on the compressive strength results of IOT cement mortar is shown in Figure 5. When the water–cement ratio increased from 0.40 to 0.45, the compressive strength at 3 days decreased from 5.7 MPa to 5.3 MPa, the compressive strength at 7 days decreased from 8.8 MPa to 7.3 MPa, and the compressive strength at 28 days decreased from 17.7 MPa to 15.8 MPa. When the water–cement ratio increased to 0.5 or even 0.55, the compressive strength at 3 d/7 d/28 d decreased to varying degrees. Water was crucial in the reaction when preparing cementitious materials from active volcanic ash materials, although it does not directly participate in the reaction; it also plays a role in dissolving alkalis and acts as a medium in the reaction [12]. When the water–cement ratio is small, the workability of the slurry is poor, and the mortar cannot fill the gaps between the aggregates well during the vibration process. When the adhesive ratio is high, water consumption is relatively high and cannot be fully converted into bound water. After mixing, bleeding and segregation occur, which affects the formation of the test block and thus the compressive strength. Within an appropriate range, there is a negative correlation between the water–cement ratio and the compressive strength of cementitious materials, indicating that reducing the water–cement ratio helps improve the compressive strength of iron tailing-based cementitious materials [6,32]. Studies have shown that IOTs mainly play a filling, nucleation, and dilution role in the cementitious material system. When IOTs are fine, they can also exhibit certain volcanic ash activity [33].

**Figure 6:**
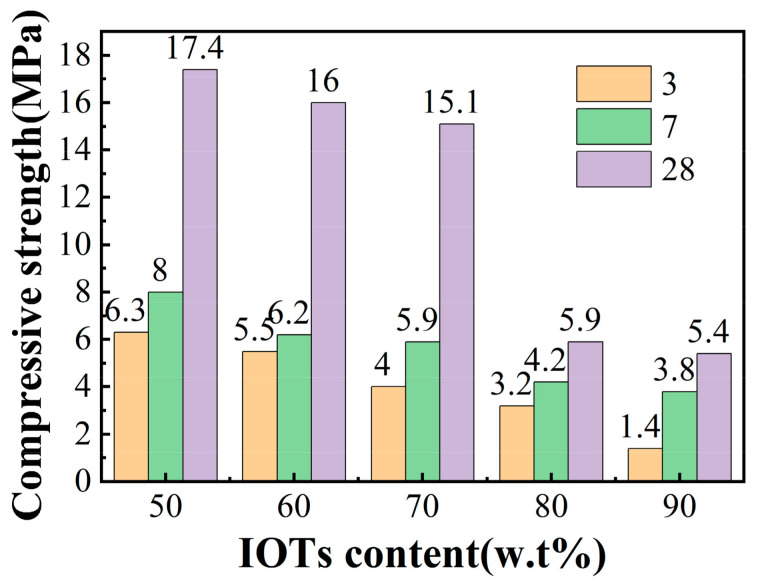
Effect of different iron tailing admixtures on the compressive strength of test blocks.

Figure 6 shows the 3-day, 7-day, and 28-day compressive strength test results of the IOT cement mortar, indicating that the compressive strength of cement mortars was significantly reduced with an increase in IOT content. This phenomenon was caused by the deficient hydration activity of IOTs. It is worth noting that the mechanical properties of the test blocks are relatively good at 50–70% content. This is because the fine IOT particles could fill and optimize internal pores, resulting in an increased matrix density. When the content of IOT increased to 80 or even 90%, the mechanical properties of the test block sharply decreased. The decrease in compressive strength can be explained as follows: (1) poor gradation increases porosity, resulting in the deterioration of mechanical properties [1] and (2) the amount of cement slurry attached to the IOT per unit area reduced, which weakens the bond strength between aggregates [34,35]. This is also confirmed in Section 3.5. Saedi et al. [36] found that with an increase in IOT content, the durability evaluation index of IOTC, including mass and strength losses, decreased. Another study also shows that the compressive strength of cement mortars reduced from 50 MPa to 12.5 MPa when IOT contents increased from 0% to 50% [37].

The iron tailing-based cementitious material was prepared according to the orthogonal test in Table 3, and the corresponding compressive strength was measured after curing at different ages. As shown in Table 5, it can be observed that the 3-day and 7-day compressive strengths of No.8 are 2.5 MPa and 5.4 MPa, respectively. Therefore, the optimum conditions are as follows: IOT content at 86%. Furthermore, we prepared specimens of different ages with the following ratios: 86% IOT, 10% GGBS, and 4% cement clinker. The reaction mechanism was analyzed via XRD, FTIR, and SEM.

### 3.3. XRD Analysis of IOT-Based Cementitious Composites

**Figure 7:**
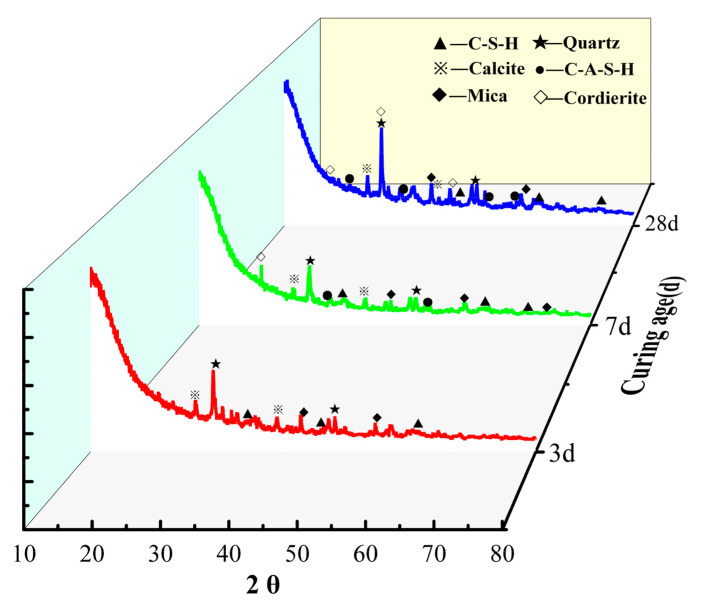
XRD analysis of iron tailing-based cementitious materials at different maintenance ages.

The XRD analysis of the 3 d, 7 d, and 28 d cementitious specimens prepared from iron tailings with slag and cement was carried out, as shown in Figure 7. The presence of a large number of dispersion fronts in the XRD pattern of the activated iron tailing-based gelling material indicated that it is mainly dominated by amorphous materials, and a wider dispersion front of amorphous materials appeared at 2θ angles of 20° to 40°, with its peak slightly shifted in the direction of the larger angle compared to the raw material, indicating that the reaction had a new production of amorphous materials mainly comprising amorphous aluminosilicate gels. This mainly resulted in composite gelling material characteristics.

In addition, the 3 d, 7 d, and 28 d specimens’ mineral compositions were essentially the same, consisting mainly of quartz, calcite, C-S-H, and C-A-S-H, indicating that their mineral phases were produced at 3 d; therefore, the early intensity at 3 d and 7 d was also greater. The small amount of quartz and calcite diffraction peaks comprised the residual minerals in the system that exhibited incomplete reactions [23,38]. Compared with the diffraction peaks at 3 d and 7 d, the quartz and calcite diffraction peaks in the 28 d specimen decreased to different degrees, and a “pack peak” formed by amorphous material appeared in the range of 20–40° at an angle of 2θ. With the extension of the maintenance age, the surface of iron tailing particles and slag particles continued to be eroded by alkali, and the area of this pack peak gradually increased. The area of the pack peak gradually increased, and the intensity of the corresponding calcite diffraction peak gradually decreased (the intensity of the corresponding calcite diffraction peak at 28 d increased instead of decreasing, and the peak area increased; this was probably due to superposition with the peaks of the generated gelling material), indicating that the quartz and clay minerals further dissolved. Moreover, the silica–alumina components were dissolved, and they were used in the hydration reaction, generating more amorphous silica–alumina gel phases and cordierites with certain strengths. All these factors collectively contribute to the enhancement of the compressive strength of the specimens.

### 3.4. FTIR Analysis of IOT-Based Cementitious Composites

**Figure 8:**
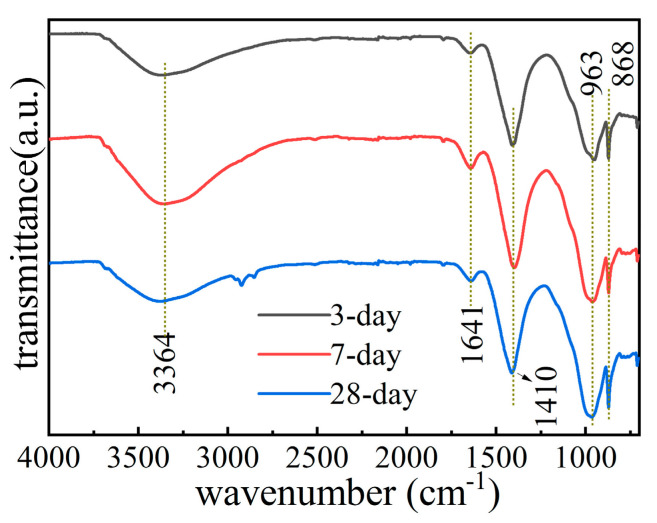
FT-IR analysis of iron tailing-based cementitious materials at different maintenance ages.

The FT-IR spectra of iron tailings obtained under different conservation age conditions are similar in Figure 8, indicating that they are products obtained by undergoing a similar chemical reaction process.

The absorption peak at 868 cm^−1^ was assigned to the superposition of the asymmetric stretching vibration of Si-O-Al [15,39]. The sharp peak at 963 cm^−1^ is due to the asymmetric Si-O-Si or O-Si-O of the SiO_4_ tetrahedral structure’s vibrations. The results indicated that new structures were generated during the preparation process—mainly dimers, trimers, and multimers formed via the polymerization of [AlO_4_] and [SiO_4_]—resulting in a complex network structure. In addition, the absorption peaks near 3364 cm^−1^ and 1641 cm^−1^ in the curves were attributed to the characteristic bands of hydroxyl stretching vibration peaks and bending vibration peaks in the water molecules contained in the iron tailings [40], which are caused by the small amount of water adsorbed in the iron tailings at the conservation age. The absorption peak near 1410 cm^−1^ corresponded to the asymmetric stretching vibration of CO_3_^2−^, which may be caused by the formation of CO_3_^2−^ products from the interaction of excess alkali excitants with CO_2_ in the air; they may also be related to the carbonates in the iron tailings. These observations, as a result of the contact carbonation of Ca(OH)_2_ formed via the reaction of small amounts of CaO and water in iron tailings, are also reported in the literature [41,42].

### 3.5. SEM Analysis of IOT-Based Cementitious Composites

**Figure 9:**
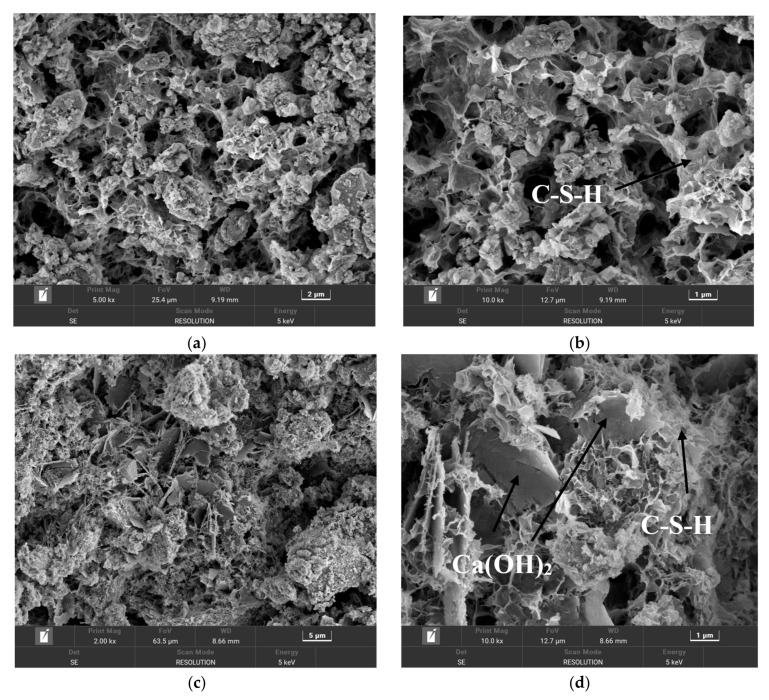

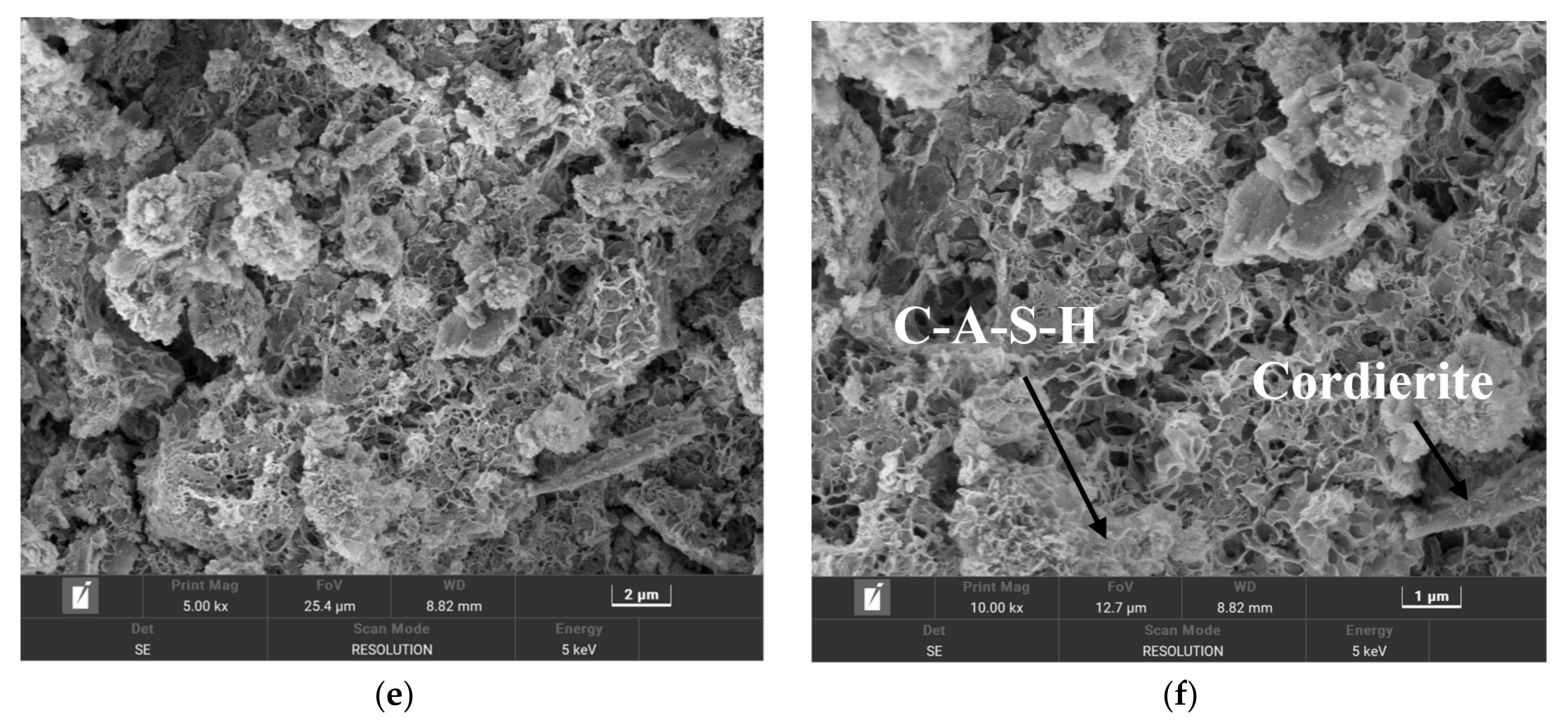
SEM images of iron tailing-based cementitious materials at different ages: (**a**) 3 days, 5000 magnification, (**b**) 3 days, 10,000 magnification, (**c**) 7 days, 2000 magnification, (**d**) 7 days, 1000 magnification, (**e**) 28 days, 5000 magnification, and (**f**) 28 days, 10,000 magnification.

The microscopic morphology of the IOTCC at different maintenance ages of 3 d, 7 d, and 28 d was analyzed, as shown in Figure 9. The microscopic morphology of the 3 d, 7 d, and 28 d gelling materials are similar. The internal structure of the dense three-dimensional network structure of the C-A-S-H gel phase (amorphous calcium silicate gel phase) and C-S-H gel phase (hydrated calcium silicate) was formed, and the C-A-S-H gel phase is the gel phase produced by the polymerization reaction of active silica–oxygen tetrahedra, alumina–oxygen tetrahedra, and alkali exciters in the iron tailings and slag. The C-S-H gel phase is the gel phase produced by the hydration reaction of silica–oxygen tetrahedra with CaO in the iron tailings under the action of alkali exciters. Many hornblende and calcite flakes can be seen more clearly with the network-like gel phase, which is glued together and dispersed inside the specimen and can be observed at a magnification of 20,000 times. At 3 d, the particles had irregular edges, many pores, loose mesh structure, low hydration, and low compressive strength.

As shown in Figure 9a, the internal pores were large at the maintenance age of 3 d because gel phases were not generated yet. At the maintenance age of 7 d (Figure 9b), it can be observed that the voids reduced, the number of voids decreased, and the number of gel phases increased. At the maintenance age of 28 d (Figure 9c), the calcite in the crystalline phase is dissolved, exhibiting a cross-section that is uniformly covered by the gel phase. The generated C S-H is stripped from the surface of iron tailing particles and moves toward the liquid phase, with some inserted into the gel structure with spatial reticulation results; this, in turn, improves the density of the material [43]. This is consistent with the presence of the diffraction peaks of some minerals in the XRD pattern in Section 3.3, in addition to the presence of a large number of amorphous noncrystalline dispersion fronts. The presence of minerals, such as quartz and calcite, which have not completely reacted, mainly plays a filling effect. With an increase in hydration products, the number of crystals that precipitate in the system increases, and crystallization and gelling overlap each other. The structural system of the specimen exhibited increased density, thus improving the mechanical properties [44].

### 3.6. Analysis of Wind Erosion Resistance Performance

The economic efficiency of MT-based geopolymer production depends on the number of costs incurred by consumers of mine waste for their transportation. In this regard, the territorial proximity of the mining enterprise relative to the planned consumers of MTs and the areas of consumption of finished geopolymer products is of significant importance. Accordingly, this experiment attempts to apply the IOTCC to dust control in the open-pit mine area. In this section, the ratios used in the above experiments are shown. Iron tailing-based crust layers were prepared and tested for dust control in open-pit mines. Various properties of the crust layer, such as the dust suppression rate, condensation time, and freeze–thaw resistance, were also investigated.

As shown in Table 6, three parallel blowing tests were conducted on the modified material. The wind erosion rate was less than 0.5%. It was observed that the IOTCC crust exhibited good dust suppression performance.

As shown in Table 7, the test preparation of two commercially available crust-type dust suppression materials was carried out. The dust suppression rate, dust suppression period, and the cracking of the shell layer were compared, and the results show a dust suppression rate of up to 99.5%. Moreover, the effective dust suppression period was more than 8 months, with basically no cracking and breaking and good dust suppression performance.

### 3.7. Water Requirement of Normal Consistency and Setting Time of the Crust Layer

The experimental data are shown in Table 8. The results show that the initial setting time was 48 min, and the final setting time was 91 min. The materials prepared in this experiment could meet the requirements of relevant standards.

### 3.8. Analysis of Freeze–Thaw Cycle Resistance

**Figure 10:**
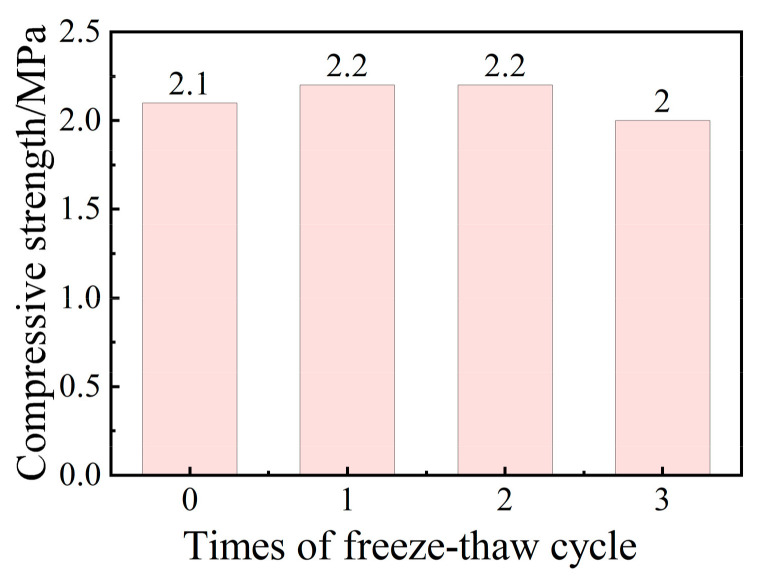
Results of the crust-layer compressive strength and the number of the F–T cycle.

As observed in Figure 10, after one freeze–thaw test, the compressive strength of the test blocks increased from the initial 2.1 MPa to 2.2 MPa. It could be considered that further hydration and hardening reactions had taken place in the system, which helped produce more gel phases in the system and improved the mechanical properties of the materials. After three freeze–thaw tests, the compressive strength of the test block was 2.1 MPa; multiple extreme conditions destroyed the structure of the already generated gelling material inside the test block. Overall, the compressive strength of this dust suppression material was affected by the freeze–thaw cycle, but only by a little as the decline was only 5%. The material exhibited good freeze–thaw resistance, which was attributed to continuous F–T cycles, and this promoted internal deformations and the internal interfacial damage of the IOTCC. The bond strength of the cement mortar decreased gradually [1].

## 4. Conclusions

In this research study, the mechanical activation effects on IOT powders’ pozzolanic activities, hydration characteristics, and applicability in cementitious materials were studied. The following conclusions were drawn from the study:

(1) With prolonged grinding times, the structural degradation of IOTs occurred, especially for the clay minerals stilpnomelane and muscovite in the IOTs. Mechanical activation could induce the amorphization of IOT powders, which was beneficial in improving the pozzolanic activity of IOTs. The D50 values of IOT powders were 5.143 μm and 4.346 μm; the D90 values of IOT powers after grinding for 20 s were obtained. The activity index of iron tailings increased from 42.3% (unground) to 78.6%;
(2) In the IOT–calcium oxide–anhydrite system, fiber-like ettringite and amorphous C-S-H gel were the major hydration products. The IOTCC was prepared by blending 86% IOT, 10% GGBS, and 4% cement clinker. When the water–cement ratio was 0.4, the compressive strength after curing for 7 days and 28 days was 8.8 MPa and 17.7 MPa, respectively;
(3) The initial setting time of the iron tailing dust suppression material was 48 min, and the final setting time was 91 min. When the material is sprayed on the tailings at a dry beach or other similar sites, it can condense and harden within more appropriate time periods to achieve the effect of dust suppression;
(4) The dust suppression material of the iron tailings’ foundation layer exhibited good stability. It exhibited good effects in conditions of wind erosion, rainfall, and freezing and thawing, and its dust suppression performance exhibited a wind erosion resistance rate of over 99.5%, with an effective period of more than 8 months. Durability, frost resistance, and high-temperature resistance were good.

Currently, IOTs are used as supplementary materials for cementitious materials, and the dosage is usually not higher than 30%, which cannot fully realize its reduction and utilization. At the same time, there are few studies on the pollution control of tailings reservoir interfaces. In this study, IOTs were used as raw materials to prepare high-volume (more than 80%) tailing-based composite cementitious materials, and they were applied to control the interface pollution of tailings reservoirs with low mechanical properties to achieve dust control in mining areas.

## Figures and Tables

**Table 1:** Chemical compositions of the raw materials (wt%).

Chemical Composition	IOT	GGBS	Clinker
Fe_2_O_3_	19.510	0.466	5.957
SiO_2_	26.113	27.070	24.765
Al_2_O_3_	7.201	19.958	14.159
CaO	23.978	31.559	43.092
MgO	18.447	14.055	3.915
TiO_2_	0.212	1.763	0.821
SO_3_	1.782	2.833	4.353
K_2_O	1.081	0.487	1.645
P_2_O_5_	0.087	0.021	0.105

**Table 2:** Proportioning for the orthogonal test.

No.	GGBS/g	Clinker/g	NaOH/g	Na_2_SiO_3_/g	IOT/wt%	W/C
1	24	4	16	8	92	0.45
2	24	12	24	16	90	0.45
3	24	20	32	24	88	0.45
4	32	4	24	24	90	0.45
5	32	12	32	8	88	0.45
6	32	20	16	16	86	0.45
7	40	4	32	16	88	0.45
8	40	12	16	24	86	0.45
9	40	20	24	8	83	0.45

**Table 3:** Effect of different water–cement ratios on the compressive strength of test blocks.

No.	W/C	W/mL	NaOH/g	Na_2_SiO_3_/g	IOT/g	GGBS/g	Clinker/g
F1	0.40	144	16	24	288	48	24
F2	0.45	162	16	24	288	48	24
F3	0.50	180	16	24	288	48	24
F4	0.55	198	16	24	288	48	24

**Table 4:** D_90_ and D_50_ of iron ore tailings at different vibratory grinding times.

Milling Time (s)	0	20	40	60	120	180	240	300	360	420
D_50_ (μm)	9.107	5.143	4.844	4.817	4.089	4.346	4.728	5.734	6.271	6.596
D_90_ (μm)	38.547	16.588	16.347	16.644	15.720	14.667	16.600	17.477	20.012	20.612

**Table 5:** Compressive strength of the orthogonal test design.

No.	IOT/wt%	W/C/%	Compressive Strength/MPa	
			7 d	28 d
1	92	0.45	0.2	0.8
2	90	0.45	0.7	2.3
3	88	0.45	0.7	2.4
4	90	0.45	1.3	3.1
5	88	0.45	0.5	2.1
6	86	0.45	1.7	4.0
7	88	0.45	1.5	3.2
8	86	0.45	2.5	5.4
9	83	0.45	2.0	3.4

**Table 6:** Results of the dust suppression rate of the crust layer.

NO.	G1/g	G2/g	υ/%	L/%
1	292.6	292.0	0.2	99.8
2	278.8	277.9	0.3	99.7
3	283.4	282.1	0.5	99.5

**Table 7:** Results of the wind erosion rate of dust suppression materials.

Dust Suppressant	L%	Dust Suppression Period	Cracking Situation
A	99.5	>8 months	No
B	92.3	>6 months	Yes
C	90.7	>2 months	Yes

**Table 8:** Results of the wind erosion rate of dust suppression materials.

Material	Water Requirement for Standard Consistency (%)	Initial Setting Time (min)	Final Setting Time (min)
Iron tailing-based cement	41	48	91
P.O42.5 cement	25.4	>45	<390

## Data Availability

Data are contained within the article.
